# Neural correlates of causal power judgments

**DOI:** 10.3389/fnhum.2014.01014

**Published:** 2014-12-22

**Authors:** Denise Dellarosa Cummins

**Affiliations:** Department of Psychology, University of Illinois at Urbana-ChampaignChampaign, IL, USA

**Keywords:** causal power, causal reasoning, causal judgment, causality, neural correlates of causality

## Abstract

Causal inference is a fundamental component of cognition and perception. Probabilistic theories of causal judgment (most notably causal Bayes networks) derive causal judgments using metrics that integrate contingency information. But human estimates typically diverge from these normative predictions. This is because human causal power judgments are typically strongly influenced by beliefs concerning underlying causal mechanisms, and because of the way knowledge is retrieved from human memory during the judgment process. Neuroimaging studies indicate that the brain distinguishes causal events from mere covariation, and also distinguishes between perceived and inferred causality. Areas involved in error prediction are also activated, implying automatic activation of possible exception cases during causal decision-making.

Causal inference is a fundamental component of cognition and perception, binding together conceptual categories, imposing structures on perceived events, and guiding decision-making. A type of causal inference that is of particular interest to decision scientists is *causal power judgment*. Causal power refers to the ability of a particular cause alone (when it is present) to elicit an effect, relative to other causes (Cheng, 1997). For example, selective serotonin-reuptake inhibitors (SSRI) may be considered more effective in alleviating depression than a placebo if greater depression alleviation is observed when an SSRI is ingested than when a placebo is ingested.

In probabilistic theories of causal judgment, causal power is assessed through metrics that integrate contingency information. One such normative metric is defined as

ΔP=(E|C)−P(E|~C)

that is, the probability of the effect occurring in the presence of the cause minus the probability of the effect occurring in the absence of the cause. (This metric is referred to as ΔP by Cheng (1997) and as *PNS* by Pearl (2000)). An extension of ΔP that normalizes the metric by means of the base rate of the effect measures the power of the candidate cause to generate or prevent the effect *relative* *to other possible causes*. Cheng (1997) defined this metric for causes that *generate* an effect as

$$P_{c}=\Delta P/1-P(E|\sim C).$$

This is equivalent to the metric defined by Pearl (2000) as *PS*. For causes that *prevent* the effect, Cheng (1997) defined causal power as

$$P_{c}=-\Delta P/P(E|\sim C).$$

The difficulty with the probabilistic approach is that human causal power judgments frequently depart from the normative values predicted by these metrics. This is because human causal power judgments are typically strongly influenced by beliefs concerning underlying causal mechanisms, and because of the way knowledge is retrieved from memory during the judgment process.

## Causal mechanisms

Causality is distinct from mere contingency or covariation. In causality, one event has the power to bring about another event. In covariation and contingency, two events are simply statistically dependent on one another. People cognize causal events differently than they do simple contingency or covariation, and this is apparent in neuro-imaging results: When viewing launching displays, significantly higher levels of relative activation is observed in the right middle frontal gyrus and the right inferior parietal lobule for causal relative to non-causal events (Fugelsang et al., 2005). Another study contrasted displays of normal causality with magic tricks that appear to violate causality and those that are surprising but do not violate causality (Parris et al., 2009). The results indicated that brain areas responsible for detecting expectancy violations in general (i.e., anterior cingulate cortex and left ventral prefrontal cortex) are not responsible for detecting causality violations. This function appears to be specific to the dorsolateral prefrontal cortex. In another study, identical pairs of words were judged for causal or associative relations in different blocks of trials. Causal judgments, beyond associative judgments, generated distinct activation in left dorsolateral prefrontal cortex and right precuneus, again substantiating the particular involvement of these areas in assessments of causality (Satpute et al., 2005).

Other research indicates that perceptual causality can be neurally distinguished from inferential causality. Inferential causality activates the medial frontal cortex (Fonlupt, 2003). Research involving callosotomy (split-brain) patients also indicates particular left hemispheric involvement (Roser et al., 2005). In contrast, perception of causality can be influenced by the application of transcranial direct stimulation to the right parietal lobe, suggesting that the right parietal lobe is involved in the processing of spatial attributes of causality (Straube and Chatterjee, 2010; Straube et al., 2011).

In short, neuroimaging studies show that the brain distinguishes causal events from non-causal events, and this distinction cannot simply be attributed to the surprising nature of non-causal event displays. It also distinguishes between perceived and inferred causality.

The importance of causal mechanism assessment looms particularly large in causal decision-making. People typically discount even strong covariation/contingency information if no plausible causal mechanism appears responsible for the covariation or contingency (Ahn et al., 1995). In a classic study by Fugelsang and Dunbar (2005), people read either plausible or implausible causal hypotheses and were shown covariation data that were either consistent or inconsistent with these hypotheses. A consistent case was one in which a plausible hypothesis was accompanied by strong covariation (high ΔP) or an implausible hypothesis was accompanied by weak covariation data (low ΔP). An inconsistent scenario was on in which a plausible hypothesis was accompanied by weak covariation data (low ΔP) or an implausible hypothesis was accompanied by strong covariation (high ΔP). The task was to estimate the effectiveness of the purported cause in bringing about the effect. The results showed quite clearly the impact of causal plausibility on behavioral judgments and neural processing. Areas associated with thinking (executive processing and working memory) were more active when people encountered data while evaluating plausible causal scenarios. Areas associated with learning and memory (caudate, parahippocampal gyrus) were activated when data and theory were consistent (plausible + strong data OR implausible + weak data). But when data and theory were *in*consistent (implausible + strong data OR plausible + weak data), attentional and executive processing areas were active (anterior cingulate cortex, prefrontal cortex, precuneus) Attentional and executive processing areas (anterior cingulate gyrus, prefrontal cortex, precuneus) were particularly active when plausible theories encountered disconfirming (weak) covariation. These results were interpreted to mean that people focus on theories that are consistent with their beliefs (plausible causal scenarios). They also attend to disconfirming data, but they do not necessarily revise beliefs in light of disconfirming data. This phenomenon is sometimes referred to as truth maintenance (Doyle, 1979) or belief revision conservatism (Kelly et al., 1997; Corner et al., 2010). Both strategies seek to maintain coherence in one’s knowledge base by minimizing changes to current belief in light of new information.

## Knowledge retrieval

Different types of knowledge are activated when reasoning from cause to effect than when reasoning from effect to cause. When reasoning from cause to effect, disablers are spontaneously activated; when reasoning from effect to cause, alternative causes are spontaneously activated. (Preventive causes in this literature are referred to as disablers.) Consider, for example, arguments of the form *“If Marilyn takes SSRI medication, then her depression will lift/Marilyn is taking SSRI medication/Therefore, Marilyn’s depression will lift”*. People’s willingness to accept such arguments is inversely proportional to the number of disablers activated in memory (factors that could prevent Marilyn’s depression from lifting even though she’s taking SSRI medication.) This effect has been observed in adults (e.g., Cummins et al., 1991; Cummins, 1995, 1997; De Neys et al., 2002, 2003; Vershueren et al., 2004) as well as children (Markovits et al., 1998; Janveau-Brennan and Markovits, 1999).

Recently, two models have been proposed to capture the impact of disablers on causal power judgments. In the first model, proposed by Cummins (2010), causal power judgments are captured by the following equation:

$$W_{c}=B(\alpha /(\alpha +\text{disablers)})$$

W_*c*_ represents the decision-maker’s estimated probability that the cause will in fact bring about the effect. B is a parameter that reflects the believability of the causal mechanism underlying the purported causal relationship. The inclusion of this parameter is motivated by ample research showing that people ignore or discount covariation information if no they can think of no plausible causal mechanism whereby the purported cause can bring about the effect (e.g., Ahn et al., 1995). In the model, if a decision-maker does not believe the two events are causally related, B = 0 and disablers are irrelevant and hence not activated in memory. Only when they believe a causal mechanism exists that empowers one event to evoke another (B = 1) do disablers become relevant.

**Figure 1:**
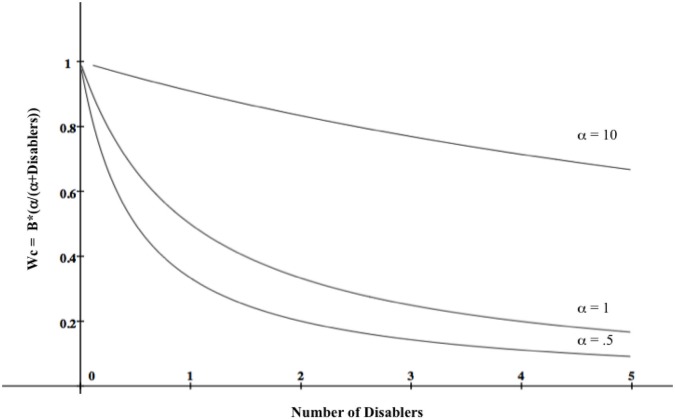
**A model of causal power values (W_*c*_) as a function of belief that a causal mechanism underlies the contingency (B) and number of disablers for different values of *α*, a free parameter whose value is determined empirically.** In the graph, B = 1, meaning that the decision-maker believes the contingency reflects a causal relationship. The function shows that the first few disablers retrieved have greater impact on causal power estimates than ones retrieved later.

The term *α*/(*α*+disablers) is a memory activation function—a positively accelerated curve—in which the first few disablers retrieved from memory have greater impact on judgment than those retrieved later. Activation spreads throughout the network of associated disablers, and likelihood estimates drop off significantly the farther it spreads. This is because stronger disablers are presumed to be activated earlier than weaker ones, and therefore have greater impact on judgment outcomes. In other words, the psychological difference between 0 and (e.g.,) 3 items is greater than the psychological difference between (e.g.,) 4 and 7. *α* is a free parameter; it simply expresses the steepness of the curve, and its value is determined empirically. Figure 1 depicts causal power likelihood estimates for different disabler and *α* values when B = 1.

The model captures the likelihood of an effect occurring when a cause is present and disablers are absent, and its crucial prediction is that the number of disablers and the order of disabler retrieval both matter.

The inclusion of *α* as a parameter is motivated by research on reasoning with causal conditional arguments. De Neys et al. (2003) reported that while “thinking aloud”, reasoners did not halt the retrieval process upon retrieving a single counterexample. Instead, they continued to retrieve disablers until a final judgment was made, and willingness to accept causal conclusions declined as more disablers were activated in memory. Their results suggested a non-linear retrieval function, however, in which a threshold occurred at about 3 retrieved items, after which argument acceptance ratings changed very little.

In the second model, proposed by Fernbach and Erb (2013), causal power judgments are based on an aggregate disabling probability. Each disabler has some prior likelihood of being present (P_*d*_) and, when present, a likelihood of preventing the effect from occurring, which constitutes its strength (W_*d*_). The disabling probability of any given disabler (A_*i*_) is equal to the product of its prior probability and its strength

$$A_{i}=P_{di}*W_{di}$$

The likelihood that the cause will successfully bring about an effect is the aggregate of these individual disabling probabilities:

$$\text{A}\prime =\sum_{i=1}^{n}{\text{A}}_{i}-\sum_{i,j:i<j}{\text{A}}_{i}{\text{A}}_{j}+\sum_{i,j,k:i<j<k}{\text{A}}_{i}{\text{A}}_{j}{\text{A}}_{k}-\ldots +{\left(-1\right)}^{n-1}\prod_{i=1}^{n}{\text{A}}_{i}$$

As an example, if there are two disablers, then the resulting equation is

$$\text{A}\prime ={\text{A}}_{1}+{\text{A}}_{2}-{\text{A}}_{1}*{\text{A}}_{2}$$

If there are three, then it becomes

$$\text{A}\prime ={\text{A}}_{1}+{\text{A}}_{2}+{\text{A}}_{3}-{\text{A}}_{1}*{\text{A}}_{2}-{\text{A}}_{1}*{\text{A}}_{3}+{\text{A}}_{1}*{\text{A}}_{2}*{\text{A}}_{3}$$

and so on. Causal power, W_**c**_, is the complement of this aggregate disabling probability, which means that it expresses the likelihood that the cause will bring about the effect when there are no disablers to prevent it:

$${\text{W}}_{c}=1-{\text{A}}^{\prime }$$

To summarize, according to Cummins (2010) (a) causal power likelihood estimates diminish as the number of disablers retrieved increases; and (b) earlier retrieved disablers have greater impact than later ones. According to Fernbach and Erb (2013), causal power likelihood can be captured by aggregate disabler impact, a value not affected by order of disabler retrieval.

Fernbach and Erb (2013) found that their model constituted a reasonably good fit for causal arguments but not for non-causal ones, despite similarity in their conditional probabilities. These results constitute strong support for the inclusion of believability parameter when modeling disabler impact. Cummins (2014) found that aggregate impact scores did not fully capture final likelihood judgments well, and the disparity was due to the fact that order of disabler retrieval mattered. Stronger disablers are retrieved first, but, contrary to Cummins’ model, the ultimate judgment is more strongly influenced by later retrieved items than by earlier ones.

Recent research has successfully identified the neurocorrelates of disabler retrieval during causal reasoning. Of particular interest are two specific event-related potentials: N2 and P3b. N2 is a frontal negative deflection observed between 200 ms and 300 ms after stimulus onset while P3b is a centroparietal positive deflection observed 250–450 ms after stimulus onset. N2 is typically observed when causal expectations are violated while P3b is typically observed when such expectations are satisfied (Verleger, 1988; Folstein and VanPetten, 2008). Causal arguments that admit of many disablers elicit more pronounced N2 and less pronounced P3b responses than do causal arguments that admit of few disablers (Bonnefond et al., 2014). This pattern of response is interpreted to mean that disabler retrieval lowers reasoners’ expectations that an effect will in fact be elicited by a particular cause.

In a related fMRI study (Fenker et al., 2010), a task cue prompted people to evaluate either the causal or the non-causal associative relationship between pairs of words. Causally related pairs elicited higher activity than non-causal associates in orbitofrontal cortex, amygdala, striatum, and substantia nigra/ventral tegmental area. Importantly, this network overlaps with the mesolimbic and mesocortical dopaminergic network known to code prediction errors (O’Doherty et al., 2003, 2007). Because the study context did not explicitly require people to make predictions, activity in this network suggests that that prediction error processing might be automatically recruited in assessments of causality.

The take-home message of this work is that human causal inference cannot be adequately modeled without taking into consideration the ways in which knowledge is activated and weighted in the decision process. Current popular models of causal inference (e.g., Fernbach et al., 2011; Fernbach and Erb, 2013) analyze it as a type of Bayesian inference, yet such models do not constitute adequate *descriptive* models of human predictive inference because they abstract away from these crucially important variables. This implies that human predictive inference is not purely Bayesian. As was well-documented by Kahneman (2011), the source of the discrepancy seems to lie in the way knowledge retrieval transacts with probability estimations. Automatic (e.g., Cummins, 1995, 2010) activation of relevant alternatives is a hallmark of human reasoning, and this characteristic must be accommodated in descriptive models of causal inference if human causal judgments are to be adequately predicted.

## Author notes

Dr. Cummins is retired from the University of Illinois at Urbana-Champaign. Correspondence regarding this research should be directed to her at denise.cummins87@gmail.com.

## Conflict of interest statement

The author declares that the research was conducted in the absence of any commercial or financial relationships that could be construed as a potential conflict of interest.
